# CoViD-19 Immunopathology and Immunotherapy

**DOI:** 10.6026/97320630016219

**Published:** 2020-03-31

**Authors:** Francesco Chiappelli, Allen Khakshooy, Gillian Greenberg

**Affiliations:** 1 Professor Emeritus, UCLA, Center for the Health Sciences, Los Angeles, CA, USA; 2 Pre-M.D. Student, Rappaport Faculty of Medicine,Technion-Israel Institute of Technology, Haifa, Israel

**Keywords:** Corona Virus Disease 2019 (CoViD-19), T cell exhaustion (Tex) markers, programmed cell death marker 1 (CD279 - PD-1), T cell immunoglobulin and mucin domain-3 (CD366 - Tim-3), cytokine storm, clinical trials

## Abstract

New evidence on the T-cell immuno-pathology in patient’s with Corona Virus Disease 2019 (CoViD-19) was reported by Diao et al. in MedRxiv (doi: 10.1101/2020.02.18.20024364) [1]. It reports observations on 522 patients with confirmed CoViD-19 symptomatology, compared to 40 control subjects. In brief, notable T cytopoenia was recorded by flow cytometry in the CD4+ and the CD8+ populations, which were significantly yet inversely correlated with remarkably increased serum levels of the pro-inflammatory cytokines IL-6, IL-10 and TNF-a. Flow cytometry established a progressive increase in the expression of programmed cell death marker-1 (PD-1) and T cell immunoglobulin and mucin domain 3 (Tim-3) as patients (n=14) deteriorated from prodromal to symptomatic CoViD-19 requiring intensive care. Here, we interpret these observations of Diao et al from our current understanding of T cell immunophysiology and immunopathology following an immune challenge in the form of sustained viral infection, as is the case in CoViD-19, with emphasis on exhausted T cells (Tex). Recent clinical trials to rescue Tex show promising outcomes. The relevance of these interventions for the prevention and treatment of CoViD-19 is discussed. Taken together, the data of Diao et al could proffer the first glimpse of immunopathology and possible immunotherapy for patients with CoViD-19.

## Background

In normal healthy subjects, about 70% of the peripheral blood lymphocytes are T cells and express the T cell receptor (TcR) in association with the cluster of differentiation # 3 (CD3). Two principal populations of T cells express either the CD4 or the CD8 moeity. CD4+CD3+ T cells recognize non-self presented by the major histocompatibility complex (MHC) class II on myeloid cells, dendritic cells and other specialized antigen-presenting cells. That step is accompanied by the production of cytokines, certain immune factors that favor immune cell proliferation. The cytokines produced at that step typically enhance the inflammatory process,and are recognized as the pro-inflammatory cytokines. They include interleukin (IL)-1b, IL-6, TNF-a, and others. Upon presentation of the nonself antigen by MHC-II, two critical binding events must occur to engage productive CD4+ cell activation, leading to proliferation aided by the pro-inflammatory cytokines and the T cell proliferation factor, IL-2, produced by the activated T cell. These binding events engage both TcR and the secondary activation site, CD28. Triggering TcR alone leads to an unproductive activation that engenders not active proliferation but a lack thereof, known as anergy. Triggering CD28 alone, signals of the CD4 cell faulty activation, and leads to programmed cell death, apoptosis.

By contrast, CD8+ T cells recognize non-self presented by MHC-I on any transformed or virally infected cell. That step is also accompanied by a pro-inflammatory cytokine storm, although the predominant ones are of the interferon family (IFN, e.g., INF-g). As for their CD4+ counterpart, CD8+ T cell activation is successful and productive when both TcR and CD28 are engaged, lest it leads to either anergy or apoptosis as outlined above.

CD4+ T cells are often recognized as the T helper population because their productive proliferation leads to the activation and maturation of B cells for the generation of antibodies to contain and neutralize bacterial and parasitic antigens. C8+ cells orchestrate the cytotoxic response to viral and cancer antigens. A third population of T cells suppress the proliferative response of activated CD4+ or CD8+ T lymphocytes, and are called regulatory T cells (Tregs). Most Tregs express the CD4 moiety, although Tregs can also be CD8+ T cells. All Tregs express the marker FoxP3. Treg-cell activation is antigen-specific, which implies that suppressive activities of Treg cells are antigen-dependent. Certain cytokines (e.g., IL-10) enhance Tregs, and therefore dampen T cell activation.

In brief, the T cell response in the normal state is a finely balanced set of events orchestrated by the three principal populations of reactive T cells: effector CD4+, effector CD8+ and FoxP3+CD4+ or FoxP3+CD8+ Tregs, and the cytokine storm they, and their corresponding antigen-presenting cells produce. Whether the activated T cells engage in productive proliferation and maturation, or unproductive anergy, or self-imposed programmed cell death is determined by certain specific receptor- and co-receptor-ligand interactions between the T cell and the antigen-presenting cell, including CD28 and its ligand. Together, these processes ensure the proper dynamic unfolding of events in a complex, yet homeostatic immunophysiologic environment. These traditional canonical immune mechanisms and other related immunoregulatory mechanisms described elsewhere [2] converge to maintain the organism in a state of immuno physiological balance. When challenged by an antigen, a specific series of coordinated heterostasis events are engaged that drive the organism to regain homeostasis. That collection of immunophysiologic adaptive events represents immune allostasis, and is characterized by laboratory evidence that point to a statistical modeling algorithm, which can be informed as an artificial model (AI) of T cell-mediated immune surveillance. The implications for the containment of epidemics by Ebola virus, Dengue virus, Zika virus, H#Na# influenza virus, as well as SARS and MERS in the context of the future immune surveillance within translational healthcare were discussed elsewhere [2]. It is timely and critical to determine if a similar AI algorithm can also be developed for CoVid-19.

Recent work in cellular immunology has uncovered that, in high-grade chronic viral infections, CD8+ T cells cannot sustain long-term activation, and enter a stage of 'exhaustion'. Not all antigenic specificities are equally prone to exhaustion, and such differential T cell exhaustion and silencing of T cell responses can be observed across hosts and even within the same host. Exhausted T cells (Tex) are epitope-specific, and different and distinct from anergic T cells. High-grade chronic viral infections may, or may not, lead to depletion of specific Tex subpopulations via apoptosis. Tex express certain specific markers, including CD279 (programmed cell death marker-1, PD-1) and CD366 (T cell immunoglobulin and mucin domain-3, Tim-3), and either produce or are modulated by certain cytokines, including IL-10. Tex cells are characterized by progressive loss of effector functions, high and sustained inhibitory receptor expression, metabolic dysregulation, poor memory and homeostatic self-renewal, and distinct transcriptional and epigenetic programs. PD-1 (CD279) and Tim-3 (CD366) expression are critical checkpoints for T cell exhaustion, as are several other biomarkers of Tex. Indeed, in vitro IL-10 blockade, or administration of IL-2, which overides PD-1 inhibitory signaling, can reverse T cell exhaustion in experimental settings. In brief, Tex is heterogeneous and includes progenitor and terminal subsets with unique characteristics and responses to checkpoint blockade [3-9].

High-grade chronic viral infections, or persistent tumor antigen stimulation results in CD8+ T cell exhaustion, which mirrors decreased effector function and proliferative capacity. Tex manifest over-expression of inhibitory receptors, including CD279 (PD-1), a lymphoid cell surface protein of the Ig superfamily, and a member of the extended CD28/CTLA-4 family of T cell regulators, which acts as a mature T cell checkpoint for the modulation of apoptosis. PD-1 interaction with either of its ligands (PD-1L1, CD274 or PD-1L2, CD273, both members of the B7 family of T cell co-receptors that includes CD28) constitute significant negative immune checkpoints in the pathway responsible for blunting cell-mediated immune responses, specifically CD8+ responses, and for upregulating resulting pathologies (e.g., CoViD-19) and malignancies [9-11].

The development of monoclonal antibodies, peptide-based and non-peptide small-molecule inhibitors of the PD-1/PD-1L pathway hold strong promise as effective beneficial immunodulators of CD8-mediated immune surveillance (9-11). Blockade of the PD-1 pathway in CD8+CD279+ subpopulation in vitro and in vivo has opened a new therapeutic avenue for abrogating functional exhaustion in these cells, and reinstating vigorous T cell cytotoxicity against the viral antigen [7-11]. Reinvegoration of Tex may also be obtained by restoring effective CD28 co-stimulation [12], since PD-1 appears to directly target CD28 cytoplasmic tail [13].

## Conclusions:

In conclusion, acquired cellular immune surveillance is a finely regulated set of processes and events that involve specific populations and sub-populations of T lymphocytes. CD8+CD3+TcR+ cells are principally responsible for the cytotoxic clearance of virally infected and tumor cells. One component of the regulatory arm of cellular immunity results in 'exhausted T cells' (Tex), which are primarily of the CD8+ population and emerge in chronic high-grade viral infection, such as what is observed in CoViD-19. Tex is broadly divided into 2 subsets: at the initial and intermediate stage, Tex^in^ are CD8+CD279^low/medium^, they retain quasi-normal mitochondrial spare respiratory capacity (SRC), and are generally responsive to PD-1 blockade of the kind outlined above. At the more advanced stage, Tex^ad^ are CD8+CD279^high^, have lower mitochondrial SRC, and are terminally exhausted and largely unresponsive to PD-1 checkpoint blockade immunotherapy. Broadly speaking, the expression of CD366 (Tim-3) in Tex parallels that of CD279 (PD-1), as well as the production of IL-10, TNF-a and other cytokines that modulate the expression of either CD279 or CD366, or both, and mediate apoptotic T cytopaenia in these patients.

In short, the evidence on Tex to date lends considerable clinical relevance to the preliminary observations reported by Diao and collaborators [1]. Their data point to significant T cytopoenia in circulating CD4+ and CD8+ T cells in patients with confirmed CoViD-19. The serum of these patients had significantly elevated levels of IL-6, IL-10 and TNF-a. Further analyses showed a progressive increase in PD-1+CD8+ and Tim-3+CD8+ subpopulation as patients deteriorated from prodromal to symptomatic CoViD-19 requiring intensive care. Taken together, the reported profile suggests the emergence of Tex in patients with confirmed CoViD-19. If those findings were to be replicated, then it would behoove clinical research scientists to develop a diagnostic protocol for CoViD-19 that include flow cytometric assessment of CD8+CD279+, and for good measure of CD8+CD366+ subpopulations. Patients with predominantly CD8+CD279^low/medium^ should be entered into vigorous immunotherapeutic protocols designed to restore CD8 cytotoxic potential by blocking the PD-1/PD-1L pathway and/or the Tim3 pathways. By contrast, palliative care only should be proffered to patients with CD8+CD366^high^, as Tex is likely overwhelming their cellular immune surveillance. It is unclear at this time whether is not the two identified subsets of Tex are differentially modulated by gonadal hormones that is to say whether or not estrogen or testosterone affect inter mediate stage or advanced stage Tex differentially or at all Tex that matter. Clinical and epidemiological observations to date indicate that men are more susceptible than women to covid-19. Data also show that the elderly are significantly more at risk to covid-19 than younger patient populations. It is possible therefore and exam problem that Tex exacerbate immuno-senescence. It is also our contention that an AI algorithm of the type described previously will be found to be relevant in the context of CoViD-19 [2]. This is of particular importance here as it could yield clinically important benefit in the immuno-pathological diagnostics of patients afflicted with CoVid-19, and directed immunotherapeutic interventions. Case in point, the specialized AI algorithm could establish the means and protocol for the restoration of T cell homeostasis from immuno-senescence and from T cell exhaustion, which should be key in the development of new and improved immuno-therapies [14], in particular for patients with CoViD-19. Case in point, the specialized AI algorithm could establish the means and protocol for the restoration of T cell homeostasis from immuno-senescence and from T cell exhaustion, which should be key in the development of new and improved immuno-therapies [14], in particular for patients with CoViD-19.

## References

[1] Diao B (2020). medRxiv preprint.

[2] Chiappelli F (2018). Bioinformation.

[3] Yi JS (2010). Immunol..

[4] Wherry EJ (2011). Nature Immunol..

[5] Saeidi A (2018). Front Immunol..

[6] Hashimoto M (2018). Annu Rev Med..

[7] McLane LM (2019). Annu Rev Immunol..

[8] Ioannidis JP (2015). PLoS Biol..

[9] Winkler  F, Bengsch B (2020). Front Immunol..

[10] Lin X (2020). Eur J Med Chem..

[11] David P (2019). Front. Immunol..

[12] Kamphorst AO (2017). Science..

[13] Hui E (2017). Science..

[14] Kasakovski D (2018). J Hematol Oncol..

